# A 5-day intensive curriculum for interns utilizing simulation and active-learning techniques: addressing domains important across internal medicine practice

**DOI:** 10.1186/s13104-018-4011-4

**Published:** 2018-12-21

**Authors:** Renee K. Dversdal, Jeffrey A. Gold, Matthew H. Richards, Joseph C. Chiovaro, Katherine A. Iossi, André M. Mansoor, Alan J. Hunter, Sima S. Desai

**Affiliations:** 1 Division of Hospital Medicine, Department of Medicine, Oregon Health & Science University, 3181 SW Sam Jackson Park Road, Portland, OR 97239 USA; 2 Division of Pulmonary & Critical Care Medicine, Department of Medicine, Oregon Health & Science University, 3181 SW Sam Jackson Park Road, Portland, OR 97239 USA; 3 Kaiser Permanente Sunnyside Medical Center, 10180 SE Sunnyside Road, Clackamas, OR 97015 USA; 4 Portland VA Medical Center, 3710 SW US Veterans Hospital Road, Portland, OR 97239 USA; 5 Division of General Medicine & Geriatrics, Department of Medicine, Oregon Health & Science University, 3181 SW Sam Jackson Park Road, Portland, OR 97239 USA

**Keywords:** Internal medicine, Residency education, Simulation, “bootcamp”

## Abstract

**Objective:**

Simulation-based learning strategies have demonstrated improved procedural competency, teamwork skills, and acute patient management skills in learners. “Boot camp” curricula have shown immediate and delayed performance in surgical and medical residents. We created a 5-day intensive, simulation and active learning-based curriculum for internal medicine interns to address perceived gaps in cognitive, affective and psychomotor domains. Intern confidence and self-perceived competence was assessed via survey before and after the curriculum, along with qualitative data.

**Results:**

A total of 33 interns completed the curriculum in 2014, 32 in 2015. Interns had a significant increase in confidence and self-perceived competence in procedural, cognitive and affective domains (all p values < .05).

**Electronic supplementary material:**

The online version of this article (10.1186/s13104-018-4011-4) contains supplementary material, which is available to authorized users.

## Introduction

Residency programs rely heavily on experiential learning for training, which may result in variable experiences between trainees and limited exposure to certain skillsets, leading to practice variation and gaps in knowledge [1]. The “boot camp” method of intensive, active learning in high density ‘blocks’ has been used with success in procedural specialties, in addition to a 3-day curriculum in internal medicine [2, 3]. We developed a 5-day bootcamp-style intern curriculum using active learning techniques with the aims of standardizing and improving performance in cognitive, affective and psychomotor domains. We hypothesized this intervention would be feasible and improve intern confidence and self-perceived competence in areas of clinical knowledge and diagnostic reasoning, as well as procedural, ultrasound, communication, and electronic health record skills.

## Main text

### Methods

The intervention commenced in September of 2014 and has continued annually since. Oregon Health & Science University Internal Medicine Residency Program is a 98-resident program with 32–33 interns/year in a tertiary referral academic medicine center. We are reporting learner self-evaluation results for the 2014 and 2015 cohorts.

The intervention consisted of a sequential 5-day intern curriculum held at the OHSU Simulation Center; 2 days progressing through the curriculum as one cohort, and 3 days rotating through six half-day stations in groups of 5–6. September was selected to allow several months of clinical experiences and context, however early enough to impact the remainder of intern year. All ‘Intensive Week’ clinical obligations were covered by upper level residents, allowing full intern participation for the 5 consecutive days of the curriculum. The program’s existing “3 + 1” (1 week of ambulatory care following every 3 weeks of inpatient clinical duties) rotation blocking minimized inpatient coverage needs, however several residents were pulled from elective blocks to cover the inpatient services [4, 5].

Curriculum domains and content were identified from informal focus groups of interns, senior, graduating, and chief residents, faculty, and program leadership. From the identified curricular needs content was developed focusing on (1) improved comfort and skills when managing urgent calls such as unstable atrial fibrillation; (2) increased communication skills during difficult conversations; (3) enhanced electronic health record (EHR) navigation and manipulation proficiency; (4) standardized procedural training for commonly performed procedures; and (5) expanded point of care ultrasound instruction for clinical assessment and procedural skills.

Additional files demonstrate the schedule for the 5-day curriculum (see Additional file 1), along with the curricular content for each session (see Additional file 2).

The primary outcomes of this study were Kirkpatrick level 1 outcomes [6] of self-perceived confidence and competence via surveys. Interns completed pre- and post-intervention surveys of baseline characteristics, prior simulation exposure, and confidence/self-perceived competence in key areas (5-point Likert scale with 5 representing the positive anchor, see Additional file 3 for anchor verbiage). Qualitative feedback was also collected.

A paired sample t-test was used to ensure similar 2014 and 2015 class characteristics. Pre/post curriculum comparisons of pooled 2014 and 2015 results were made using student paired t-tests. Cohort data are shown as mean Likert score ± standard deviation, with associated *p* values. A Chi square test was used to compare pre and post replies of “average” or above. A *p* value < .05 was considered statistically significant for all analyses. Data were analysed using the IBM SPSS statistical package version 24 (IBM Corporation, Armonk New York, USA).

This study was approved by the Oregon Health & Science University Institutional Review Board (IRB). All participants were provided a consent information sheet, and informed consent was implied by proceeding to the survey. This was approved in lieu of written or verbal consent by the IRB.

### Results

Intern participation in the intervention and completion of the pre-intervention survey was 100% for both 2014 and 2015 intern classes (N = 33 for 2014 and N = 32 for 2015). Post-intervention survey completion rates were 96% overall with 100% for 2014 and 91% in 2015. Over 85% of interns endorsed some simulation exposure in medical school. Baseline characteristics were similar with the exception of the 2014 cohort having significantly more women, and less undergraduate experience with standardized patients to teach clinical skills (see Additional file 4). Because of similarities in class size, participation, and overall demographics we pooled analysis for the 2 years (see Additional file 5).

Approximately 14 faculty were involved to varying degrees each year, with a total of 96 faculty hours required for days 1–2 and 216 faculty hours for days 3–5.

For procedurally-related activities participants significantly improved confidence and self-perceived competence across all procedures (Table 1). Specifically, the fraction of interns who had at least “average” self-perceived confidence across the procedures increased from 32.3 to 85.7% (p < .001). Similar results were observed with self-perceived competence (30.6 vs 81.3%; p < .001).

**Table 1:** Procedural confidence and self-perceived competence, pre and post-intervention, as measured by 1–5 Likert scale

	Confidence			Self-perceived competence		
	Mean pre	Mean post	*p* value	Mean pre	Mean post	*p* value
ABG	1.98 ± .827	3.31 ± .799	< .0005	1.93 ± .769	3.02 ± .827	< .0005
PIV placement	2.40 ± .954	3.40 ± .748	< .0005	2.41 ± .859	3.14 ± .868	< .0005
CVC placement	1.78 ± .817	3.29 ± .773	< .0005	1.86 ± .769	3.09 ± .801	< .0005
Lumbar puncture	1.90 ± .810	2.91 ± .864	< .0005	1.91 ± .823	2.86 ± .888	< .0005
Paracentesis	2.50.996	3.67 ± .825	< .0005	2.43 ± .975	3.34 ± .870	< .0005
Thoracentesis	1.78 ± .918	3.16 ± .812	< .0005	1.90 ± .852	3.02 ± .848	< .0005

**Fig. 1:**
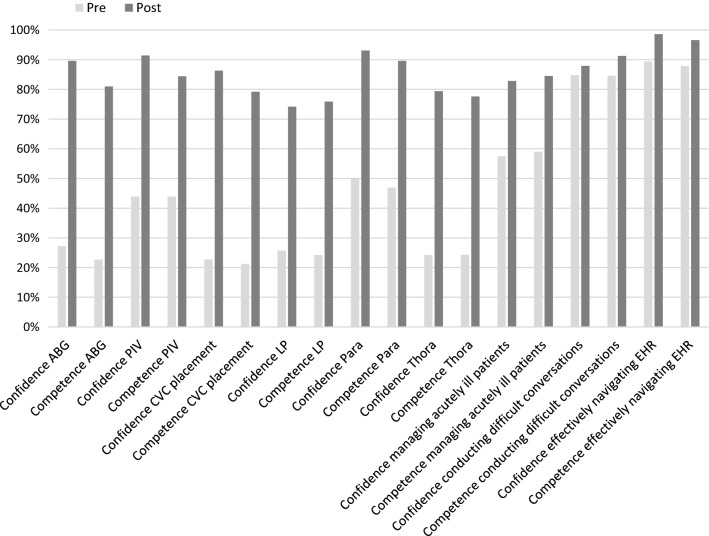
Percentage of interns self-assessing average or better. Pre and post-curriculum self-assessments of average or better, broken out by specific skill

In a similar fashion, we demonstrated an increase in confidence and self-perceived competence across the cognitive and affective domains as well (Table 2); the percentage of interns who had at least “average” self-perceived confidence increased from 77.3 to 89.7% (p < .001). Parallel results were observed with self-perceived competence (77.2 vs 90.8%; p < .001). Figure 1 illustrates the percentages of learners with self-assessments of average or better, broken out by specific skill.

**Table 2:** Cognitive and affective skills confidence and self-perceived competence, pre and post-intervention, as measured by 1–5 Likert scale

	Confidence			Self-perceived competence		
	Mean pre	Mean post	*p* value	Mean pre	Mean post	*p* value
Managing acutely ill patients	2.57 ± .752	3.02 ± .737	< .0005	2.66 ± .762	2.98 ± .635	< .0005
Conducting difficult conversations	3.28 ± .854	3.52 ± .843	.029	3.21 ± .861	3.46 ± .734	.018
Effectively navigating EHR	3.34 ± .849	3.69 ± .799	.001	3.24 ± .802	3.55 ± .799	.002

Final evaluation feedback was positive: 97% of interns in the included classes felt the curriculum was a good use of their time, 94% felt it should be continued (.02% no, .046% unsure), and 89% felt it should be expanded across the second and third years of residency (11% unsure, 0% no). Team building and developing camaraderie with colleagues were unforeseen benefits described by interns when debriefing the experience with the residency Program Director.

### Discussion

In our study, we demonstrated that an annual, week-long, “boot-camp” type curriculum, incorporating active learning techniques such as simulation, is feasible to create and reproduce. This curriculum, employing high-fidelity case-based simulations, procedural simulation with task trainers, communication simulations with standardized patients, and electronic health record (EHR) simulated exercises demonstrated improved intern confidence, and self-perceived competence in performing tasks in cognitive, affective and psychomotor domains.

This curriculum is unique in several ways. Instead of a true “boot-camp” occurring prior to internship, such as the 3-day Northwestern University simulation-based mastery learning curriculum for internal medicine interns [3], our curriculum occurs several months into the year allowing time to gain clinical context within our health care system.

In addition, the central focus of the Northwestern curriculum was on the summative assessment of learning. In contrast many simulation-based activities revolve around formative assessment which has been described as an assessment for learning rather than of, and is intended to shape skills and knowledge through feedback, highlighting, and closing performance gaps [7]. Other than ours, there are to date no published internal medicine intensive simulation programs which focus solely on formative feedback and skill development. Finally, the 5-day timeframe allowed us to include an increased scope of skills and materials versus the majority of published graduate medical education which focus on surgical and procedural skills instead of communication or collaborative skills [2], even the more comprehensive Northwestern Internal Medicine curriculum. We included ultrasound, EHR, and clinical skills training relevant to care ranging from outpatient to acute care settings. We feel this is crucial given the wide breadth of knowledge and skills required in internal medicine practice.

Of note, we were able to demonstrate improvements in intern confidence and self-perceived competence despite the intervention occurring later in the year than other documented “bootcamps”. Even starting in September, almost 3-months into the year, we were able to increase the overall number of learners meeting a threshold of at least average confidence and self-perceived competence. This would suggest that similar curricula might benefit trainees throughout the year, and could allow for increasing complexity in topic, technical skills, and concepts.

One consideration to reproducing these findings is the amount of resources required. While we use our Simulation Center, many activities could be run in situ or in classrooms. The most cost-limiting resource may be the faculty, if faculty are not volunteering their time. The current strategy necessitates a minimum of six faculty members over 5 days. This required approximately 14 faculty per year, for a total of 312 h. There is also a significant time commitment in training faculty to participate; which decreases with return faculty. In addition to protected time for interns, the faculty time requires a substantial departmental commitment.

Further analysis is needed to assess the impact of such curricula with escalating complexity and difficulty in more senior residents or faculty. In addition, future studies could determine if the effects on confidence and self-perceived competence are sustained, or if self-perceived competence correlates with demonstrable competence or clinical outcomes.

### Limitations

- There was no randomization or control group; interns served as their own historical controls.
- Intern confidence and perceived competence were assessed, however there were no formal competency assessments.
- While we improved self-perceived competency during this activity, we did not assess objective clinical outcomes.

## Additional files

**Additional file 1.** “Example Schedule”. Sessions in light grey were conducted in a large group with small group breakouts, and those in dark grey were conducted in rotating small groups.

**Additional file 2.** “Curricular content for each session”. This provides more in-depth descriptions of each session.

**Additional file 3.** “Likert values from surveys” which lists the Likert anchor verbiage.

**Additional file 4.** “Demographics & Baseline Experience of Intensive Week Interns Separated by Year”. This includes standard demographics plus United States Medical Licensing Examination (USMLE) scores and previous experiences.

**Additional file 5.** “Demographics & Baseline Experience of Intensive Week Interns, 2014 + 2015 cohorts”. This includes standard demographics plus United States Medical Licensing Examination (USMLE) scores and previous experiences.

## Abbreviations

ABG: arterial blood gas
CVC: central venous catheter
EHR: electronic health record
IRB: Institutional Review Board
LP: lumbar puncture
OHSU: Oregon Health & Science University
Para: paracentesis
PIV: peripheral IV
Thora: thoracentesis
