# Supplementary material for: A 5-day intensive curriculum for interns utilizing simulation and active-learning techniques: addressing domains important across internal medicine practice
Source: BMC Res Notes. 2018 Dec 21;11:916. doi: 10.1186/s13104-018-4011-4 (PMC6302521; doi:10.1186/s13104-018-4011-4)
Supplement: Supplementary file 1 — Additional file 1. “Example Schedule”. Sessions in light grey were conducted in a large group with small group breakouts, and those in dark grey were conducted in rotating small groups. [file 13104_2018_4011_MOESM1_ESM.docx]

**Additional file 1**: Example Schedule

|  | **Day 1** | **Day 2** | **Day 3** | **Day 4** | **Day 5** |
| --- | --- | --- | --- | --- | --- |
|  |  |  | **Rotation 1** | **Rotation 2** | **Rotation 3** |
| **8am-12pm** | **The Essentials** | **Ultrasound Training** | **Communication Skills** | **On Call Crisis Management 1** | **Central Venous Lines** |
|  |  |  | **Rotation 2** | **Rotation 4** | **Rotation 6** |
| **1pm-5pm** | **Needles 101*** | **Ultrasound Training** | **EHR applied cases** | **Thora & Para** | **On Call Crisis Management 2** |

*In 2015 “Needles 101” was moved to the morning with “The Essentials” and another half-day ultrasound session was added.
