# Supplementary material for: A 5-day intensive curriculum for interns utilizing simulation and active-learning techniques: addressing domains important across internal medicine practice
Source: BMC Res Notes. 2018 Dec 21;11:916. doi: 10.1186/s13104-018-4011-4 (PMC6302521; doi:10.1186/s13104-018-4011-4)
Supplement: Supplementary file 2 — Additional file 2. “Curricular content for each session”. This provides more in-depth descriptions of each session. [file 13104_2018_4011_MOESM2_ESM.docx]

**Additional file 2:** Curricular content for each session

**“On-Call Crisis Management” Session**

Curriculum for the “on-call crisis management” was developed by creating or adapting(1) simulations utilizing interactive computer-driven patient simulator. This allowed interns to work through diagnostic and management decisions for common acute conditions. The same faculty “champion” was available for each session with an additional faculty member if available. The experience and cognitive materials surrounding the case were discussed during a debriefing session immediately after each case. A formal debriefing tool was not utilized, rather a general advocacy-inquiry approach(2). Some of these sessions included peer feedback and peer teaching.

**Communication Session**

Addressing strong emotion and facilitating code status conversations were identified as targets in the Communication session. Short didactic sessions introduced the tools Ask-Tell-Ask, NURSE, and SPAM to aid in giving bad news, addressing emotion, and guiding code status conversations respectively(3,4). Most of the session was devoted to role play with the resident delivering serious news to a patient’s family member, played by a trained actor, in the Vital Talk method of simulation that one of our local leaders was trained in (www.vitaltalk.org)(5). Facilitators encouraged learners to try new skills and guided resident observers to give specific, behavioral feedback. Residents also participated in role plays of common code status scenarios with each other and recorded skills they wanted to continue practicing in a letter to themselves that was mailed to them six months later. Residents were surveyed pre and post-intervention regarding the effect the training had on their perceived communication skills.

**“The Essentials” Session**

“The Essentials” consisted of a lecture on radiographic confirmation of various line placements along with procedural complications, followed by role playing of sterile & non-sterile technique, discussions about potential complications of procedures, along with appropriate actions. “Needles 101” consisted of rotations through 6 stations including ABG, PIV placement, lumbar puncture, nasogastric tube insertion, knee arthrocentesis, and suturing on pigs’ feet. In 2015 these sessions were combined into one half-day consisting of the radiology lecture followed by “Needles 101” stations to reduce inactive time for the learners and allow additional ultrasound training. Six facilitators were required for the “Needles 101” portion.

**Central line placement Session**

Central line (CVC placement) training consisted of proceeding through four published University of Washington CVC Project simulation stations(6). These sessions were facilitated by two faculty or fellows from anesthesia, cardiology, or critical care.

**Paracentesis & Thoracentesis Session**

The paracentesis and thoracentesis station was led by one faculty member competent in both procedures. Each session began with a white board review of the cognitive aspects of thoracentesis, including indications, contraindications, troubleshooting, and complications. Every step of the procedure from consent to documentation was reviewed. A complete kit was utilized to allow visualization of the key items. Finally, each intern applied the reviewed concepts via practice on a task trainer. The faculty member observed and coached until both intern and faculty member were satisfied with technique. The same process was then repeated for paracentesis.

**Electronic Health Record Session**

The Electronic Health Record (EHR) portion of the Intern Intensive curriculum was developed to facilitate the intern’s abilities with the Oregon Health & Science University EHR, EpicCare, and was run by a single faculty member. EHR curricular details and detailed results on EHR usability from the 2014 session were previously published(7).

**Point of Care Ultrasound Session**

The Point of Care ultrasound curriculum consisted of one day in the first year of the curriculum, one and one-half days subsequently. Six volunteer instructors were required, and training consisted of alternating didactics with small group hands-on sessions, which have been described elsewhere(8).

**References**

1. Sargsyan Z, Kohn R, Alba G, Heath J, Currier P, Hayden E, et al. Simulation Curriculum in Internal Medicine: Decision-Making Training for Interns Focusing on Acute Clinical Scenarios (10 Cases for Fall). MedEdPORTAL Publ.

2. Rudolph JW, Simon R, Dufresne RL, Raemer DB. There’s no such thing as "nonjudgmental" debriefing: a theory and method for debriefing with good judgment. Simul Healthc. 2006;1(1):49–55.

3. White J, Fromme EK. "In the beginning...": tools for talking about resuscitation and goals of care early in the admission. Am J Hosp Palliat Care. 2013 Nov 1;30(7):676–82.

4. Responding to Emotion: Respecting [Internet]. [cited 2017 Aug 1]. Available from: http://vitaltalk.org/guides/responding-to-emotion-respecting/

5. Back AL, Arnold RM, Tulsky JA, Baile WF, Fryer-Edwards KA. Teaching communication skills to medical oncology fellows. J Clin Oncol. 2003 Jun 15;21(12):2433–6.

6. Figueredo E, Sinanan M, Makarewicz V, Kim S, Wright A. Improving Patient Safety and Reducing Hospital Costs: The University of Washington Central Venous Catheter Project - Healthcare Training and Education [Internet]. Healthcare Training and Education. 2012 [cited 2017 Jan 31]. Available from: https://www.healthcaretrainingandeducation.com/university/improving-patient-safety-and-reducing-hospital-costs-the-university-of-washington-central-venous-catheter-project/

7. March CA, Scholl G, Dversdal RK, Richards M, Wilson LM, Mohan V, et al. Use of Electronic Health Record Simulation to Understand the Accuracy of Intern Progress Notes. J Grad Med Educ. 2016 May;8(2):237–40.

8. Clay RD, Lee EC, Kurtzman MF, Dversdal RK. Teaching the internist to see: effectiveness of a 1-day workshop in bedside ultrasound for internal medicine residents. Crit Ultrasound J. 2016 Dec 11;8(1):11.
