# Supplementary material for: A 5-day intensive curriculum for interns utilizing simulation and active-learning techniques: addressing domains important across internal medicine practice
Source: BMC Res Notes. 2018 Dec 21;11:916. doi: 10.1186/s13104-018-4011-4 (PMC6302521; doi:10.1186/s13104-018-4011-4)
Supplement: Supplementary file 3 — Additional file 3. “Likert values from surveys” which lists the Likert anchor verbiage. [file 13104_2018_4011_MOESM3_ESM.docx]

**Additional file 3**:

Likert values from surveys

|  | For Confidence Questions | For Self-Perceived Competence Questions |
| --- | --- | --- |
| 1 | Not at all confident | Not at all competent |
| 2 | Below average confidence | Below average competence |
| 3 | Average confidence | Average competence |
| 4 | Above average confidence | Above average competence |
| 5 | Very confident | Very competent |
