# Supplementary material for: A 5-day intensive curriculum for interns utilizing simulation and active-learning techniques: addressing domains important across internal medicine practice
Source: BMC Res Notes. 2018 Dec 21;11:916. doi: 10.1186/s13104-018-4011-4 (PMC6302521; doi:10.1186/s13104-018-4011-4)
Supplement: Supplementary file 4 — Additional file 4. “Demographics & Baseline Experience of Intensive Week Interns Separated by Year”. This includes standard demographics plus United States Medical Licensing Examination (USMLE) scores and previous experiences. [file 13104_2018_4011_MOESM4_ESM.docx]

**Additional file 4**: Demographics & Baseline Experience of Intensive Week Interns Separated by Year

|  | 2014 Cohort | 2015 Cohort | *p* value |
| --- | --- | --- | --- |
| Age | 28.9 | 27.6 | 0.035 |
| Percent Female | 71 | 23 | <.0005 |
| Average Step 1 USMLE* | 243.5 | 244.9 | 0.734 |
| Average Step 2 USMLE* | 249.7 | 258.7 | 0.049 |
| Medical School Simulation Experience | 85% | 88% | 0.560 |
| Medical School Standardized Patient Experience for Teaching Clinical Skills | 88% | 100% | 0.045 |
| Medical School Standardized Patient Experience for Assessing Clinical Skills | 91% | 100% | 0.085 |
| Prior Ultrasound Training | 29% | 28% | 0.908 |
| Prior Epic Experience | 71% | 81% | 0.312 |
| Anticipate Performing Procedures Post-Residency | 88% | 94% | 0.436 |

*United States Medical Licensing Examination
