# Supplementary material for: A 5-day intensive curriculum for interns utilizing simulation and active-learning techniques: addressing domains important across internal medicine practice
Source: BMC Res Notes. 2018 Dec 21;11:916. doi: 10.1186/s13104-018-4011-4 (PMC6302521; doi:10.1186/s13104-018-4011-4)
Supplement: Supplementary file 5 — Additional file 5. “Demographics & Baseline Experience of Intensive Week Interns, 2014 + 2015 cohorts”. This includes standard demographics plus United States Medical Licensing Examination (USMLE) scores and previous experiences. [file 13104_2018_4011_MOESM5_ESM.docx]

Additional file 5

|  | 2014 + 2015 Cohorts |
| --- | --- |
| Age | 28.3 |
| Percent Female | 48.4% |
| Average Step 1 USMLE* | 244.2 |
| Average Step 2 USMLE* | 254.1 |
| Medical School Simulation Experience | 84.8% |
| Medical School Standardized Patient Experience for Teaching Clinical Skills | 93.9% |
| Medical School Standardized Patient Experience for Assessing Clinical Skills | 95.5% |
| Prior Ultrasound Training | 28.8% |
| Prior Epic Experience | 75.8% |
| Anticipate Performing Procedures Post-Residency | 90.9% |

*United States Medical Licensing Examination
